# Early-Stage Detection of Biotic and Abiotic Stress on Plants by Chlorophyll Fluorescence Imaging Analysis

**DOI:** 10.3390/bios13080796

**Published:** 2023-08-08

**Authors:** Julietta Moustaka, Michael Moustakas

**Affiliations:** Department of Botany, Aristotle University of Thessaloniki, 54124 Thessaloniki, Greece; ioumoustaka@gmail.com

**Keywords:** photosynthetic heterogeneity, photosystem II (PSII), reactive oxygen species, excess excitation energy, photosynthetic efficiency, redox state of quinone A (Q_A_), photochemistry, open PSII reaction centers

## Abstract

Most agricultural land, as a result of climate change, experiences severe stress that significantly reduces agricultural yields. Crop sensing by imaging techniques allows early-stage detection of biotic or abiotic stress to avoid damage and significant yield losses. Among the top certified imaging techniques for plant stress detection is chlorophyll *a* fluorescence imaging, which can evaluate spatiotemporal leaf changes, permitting the pre-symptomatic monitoring of plant physiological status long before any visible symptoms develop, allowing for high-throughput assessment. Here, we review different examples of how chlorophyll *a* fluorescence imaging analysis can be used to evaluate biotic and abiotic stress. Chlorophyll *a* is able to detect biotic stress as early as 15 min after *Spodoptera exigua* feeding, or 30 min after *Botrytis cinerea* application on tomato plants, or on the onset of water-deficit stress, and thus has potential for early stress detection. Chlorophyll fluorescence (ChlF) analysis is a rapid, non-invasive, easy to perform, low-cost, and highly sensitive method that can estimate photosynthetic performance and detect the influence of diverse stresses on plants. In terms of ChlF parameters, the fraction of open photosystem II (PSII) reaction centers (q*p*) can be used for early stress detection, since it has been found in many recent studies to be the most accurate and appropriate indicator for ChlF-based screening of the impact of environmental stress on plants.

## 1. Introduction

Global climate change has quickly turned into a huge issue in the agricultural industry due to increased episodes of drought and elevated temperature that, together with the longer sunlight hours of the Mediterranean area, detrimentally influence crop production [1,2,3,4]. For example, leguminous lentil in the Mediterranean region is affected by huge fluctuations in seasonal precipitation, with intensive rainfalls in winter, and drought and high-temperature stress from March to May [5,6]. Plants experience drought stress when the water content of the soil is limited or when transpiration is intensive [6,7,8,9]. Water-deficit stress disturbs osmotic adjustment of plants and impairs photosynthesis and growth [10,11,12], resulting, e.g., in 21% yield reductions in wheat and even in 40% in maize [13]. Drought is the principal problem in all environmental situations connected with climate change and significantly reduces global crop production [14,15]. Water deficit impairs plant cell division, elongation and differentiation, decreasing photosynthetic rates and growth, disturbing energy balance, and ultimately decreasing plant productivity [10,16,17,18]. Drought and soil compaction usually occur simultaneously and several studies revealed that the concurrent act of both causes greater effects [19]. At the same time, climate change is affecting plant–herbivore interactions. For example, higher temperatures, increased CO_2_ levels and drought stress may increase the consumption of plant tissue by herbivores and alter the development of insects [20]. Moreover, there is increasing pesticide resistance including to insecticides [21], herbicides [22] and fungicides [23], resulting in reduced crop yields [24].

Photosynthesis is vital to plant growth, functioning and fitness, but the plant’s ability to obtain and maintain high photosynthetic function significantly relies upon biotic and abiotic stress conditions [25,26]. Photosynthesis of food crops under environmental stress conditions, in order to meet the vast demand for food, is a true challenge for crop breeders and plant scientists [27,28,29]. 

## 2. The Light Reactions of Photosynthesis

**Figure 1:**
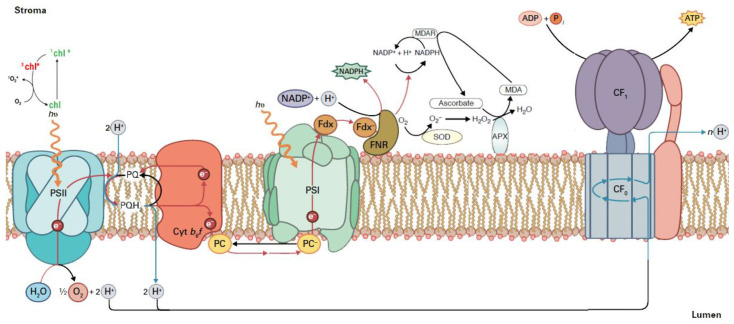
Absorption of light energy from sunlight and its conversion to chemical energy. Electrons are transferred from PSII to PSI and finally to ferredoxin (Fdx) to form NADPH. Under excess light energy, ^1^O_2_ is formed via ^3^chl*. At PSII in the water-splitting complex, the oxidation of water results in molecular oxygen (O_2_), protons (H^+^), and electrons (e^−^). The e^−^ are transferred from H_2_O to NADP^+^, and associated with this electron transfer, a proton gradient is established that is used for ATP synthesis by ATP synthase. Plastoquinone (PQ) accepts two electrons from H_2_O oxidation and two protons (H^+^) from the stroma of the chloroplast and is reduced to plastoquinol (PQH_2_), while the e^−^ are transferred to PSI through cytochrome b6f and plastocyanin (PC). A proton gradient from this electron transport is established that results in ATP synthesis. Ferredoxin-NADP+ reductase (FNR) and Fdx are also depicted. When NADP+ is not present (due to unused NADPH for carbohydrate synthesis), the superoxide anion (O_2_•−) is formed from the electrons that are transferred to O_2_. Successively, O_2_•− is converted to hydrogen peroxide (H_2_O_2_) by the superoxide dismutase (SOD), and then H_2_O_2_ is reduced to water by ascorbate peroxidase (APX). The oxidized ascorbate is reduced from NADPH through monodehydroascorbate reductase (MDAR); as a consequence, NADP+ is available (adopted from [32]).

Chlorophylls are the main molecules that absorb light energy via light-harvesting complexes (LHCs) [30,31]. In the light reactions of photosynthesis, energy is transferred from photosystem II (PSII), through cytochrome b6f and plastocyanin (PC), to photosystem I (PSI) [30,31] (for details see Figure 1). Photosystem II (PSII) is a large pigment–protein complex responsible for the light-dependent oxidation of water to molecular oxygen in photosynthetic organisms.

The products of the light reactions, ATP and NADPH, must be coordinated with the synthesis of carbohydrates and other essential organic molecules; otherwise, reactive oxygen species (ROS) are generated [31,32,33,34] (Figure 1). The absorbed light from the LHCs provides energy to a number of fundamental photosynthetic processes such as H_2_O oxidation and electron transport, coupled with the pumping of protons across the thylakoid membranes for the synthesis of ATP by ATP-synthase (utilizing the generated proton gradient) and NADPH [31,32,34] (see Figure 1).

During the process of light energy conversion to chemical energy, reactive oxygen species (ROS) are continuously formed at low levels, but they are scavenged by different antioxidant mechanisms [34,35,36,37]. These ROS are the singlet-excited oxygen (^1^O_2_), hydrogen peroxide (H_2_O_2_), and superoxide anion radical (O_2_•−) [34,35,36,37] (see Figure 1). Excess light energy absorption can definitely oversaturate the electron transport chain capacity, leading to the increased probability of ROS formation [31,34]. Biotic and abiotic stresses, such as drought, salinity, metal toxicity, chilling, UV-B radiation, insects, and pathogens result in an increase in ROS (H_2_O_2_, O_2_•−, ^1^O_2_, OH•) creation in plants, due to disruption of cellular homeostasis that can result in oxidative stress [34,38,39,40,41,42]. Oxidative stress results from an imbalance between ROS production and scavenging by enzymatic and non-enzymatic antioxidants [34,42,43,44,45]. This imbalance causes cellular damage that can lead to cell death [46,47,48,49]. Thus, the plant needs to respond to this imbalance before destruction of cellular structures to maintain photosynthetic activity and whole-plant survival [44,49]. 

Chloroplasts are the most significant creators of ROS in plant cells and especially the light reactions of photosynthesis [42]. Under most environmental stress conditions, the absorbed light energy is in surplus of what it can be controlled, and thus it can harm the chloroplast [34,42]. The process that protects the photosynthetic apparatus from the excess light energy that results in ROS generation is the mechanism of non-photochemical quenching (NPQ) [31,34,50,51]. Plants have developed several photoprotective mechanisms, including light escaping through leaf and chloroplast movement, the NPQ mechanism through dissipation of absorbed light energy as thermal energy, cyclic electron transport around PSI, the photorespiratory pathway and ROS-scavenging systems [44,51]. NPQ creation can avoid the increase in ROS generation that is often observed under environmental stress conditions [34,51,52,53]. However, the increase in ROS generation under environmental stress conditions can be eliminated by the enzymatic and non-enzymatic antioxidant mechanisms [18,34,42,47,54,55]. 

The method of chlorophyll *a* fluorescence analysis is of high resolution, rapid, non-destructive, low cost, and can evaluate any changes in photochemistry by monitoring the chlorophyll fluorescence emission of PSII [56,57,58,59,60]. This method can accurately determine the amount of energy that is used for photochemistry (Φ*_PSΙΙ_*), dissipated as heat (Φ*_NPQ_*), or non-regulated dissipated in PSII (Φ*_NO_*) [60,61,62]. 

## 3. The Basics of Chlorophyll *a* Fluorescence Analysis

Chlorophyll *a* fluorescence arises from absorbed light energy and can be interpreted as a measure of photosynthetic activity, providing valuable insights into the photosynthetic apparatus, particularly PSII [57,59,60,61]. Absorption of light energy by a chlorophyll molecule (Chl) converts it into an excited state (Chl*), with higher energy that depends on the light wavelength used for illumination [31,50]. Excited chlorophyll molecules (Chl*) can exist in two excited states: singlet-state chlorophyll molecules (^1^Chl*) that are relatively short lived, with opposite electron (antiparallel) spins, and the more long-lived triplet-state chlorophyll molecules (^3^Chl*), with electron spins that are aligned (parallel) [31,34,42,50]. Transitions from ^1^Chl* to ^3^Chl* can occur, when ^1^Chl* is not de-excited, with ^3^Chl* remaining excited for longer [31,34,50].

**Figure 2:**
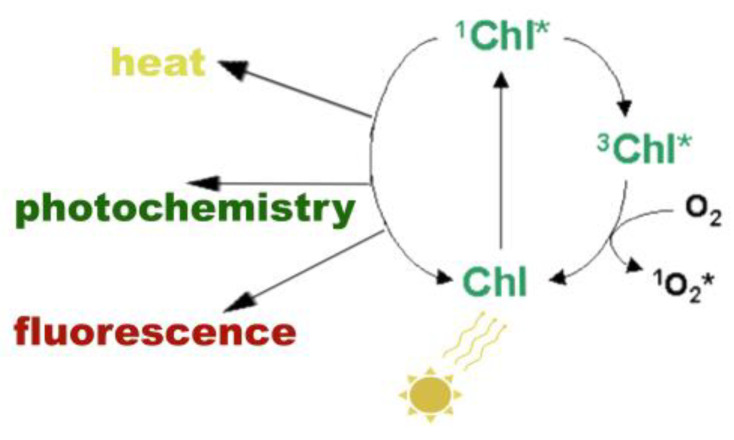
Possible pathways of singlet-excited state chlorophyll molecule (^1^Chl*) de-excitation. When a chlorophyll molecule (Chl) absorbs light energy, it converts into ^1^Chl*. From there, it has several pathways to de-excite and return back to the ground state. ^1^Chl* can be de-excited either (i) by losing energy as **heat**, (ii) by transferring the energy to another molecule that can be de-excited by losing an electron to an electron-acceptor molecule, which is called **photochemistry**, or (iii) by re-emitting light through **fluorescence**. Τhe more rapid pathway available for de-excitation of ^1^Chl* is that of photochemistry. In cases where ^1^Chl* is not de-excited by the pathways described above, it is converted from the higher-energy excited state ^1^Chl* to the lower-energy excited state ^3^Chl* by internal conversions or relaxations. ^3^Chl* can react with molecular O_2_ to produce single oxygen (^1^O_2_*), which is a very reactive oxygen species (ROS).

The singlet-excited state chlorophyll molecule (^1^Chl*) (Figure 2) can be de-excited either (i) by losing energy as **heat** (referred as NPQ), (ii) by transferring the energy to another molecule (usually nearby to the excited molecule), that finally is de-excited losing an electron to an electron-acceptor molecule, called **photochemistry** (referred as q*p*), or (iii) by re-emitting light from the lowest excited state through **fluorescence** in a longer wavelength than the absorbed light [31,50]. Among these pathways, the more rapid pathway available for de-excitation of ^1^Chl* is that of photochemistry, which converts the light energy into chemical products [31,50]. When photosynthesis proceeds with high efficiency, then little fluorescence is observed [59,60,61]. In cases where ^1^Chl* is not de-excited by the pathways described above, it is converted from the higher-energy excited state ^1^Chl* to the lower-energy excited state ^3^Chl* by internal conversions or relaxations [31,32,50]. Triplet-state chlorophyll molecules (^3^Chl*) can react with molecular O_2_ to produce single oxygen (^1^O_2_*), a very reactive oxygen species (ROS) [31,32,34,50]. At ambient temperatures, most fluorescence comes from chlorophyll *a* molecules associated with PSII [59].

## 4. Measuring Chlorophyll *a* Fluorescence

**Figure 3:**
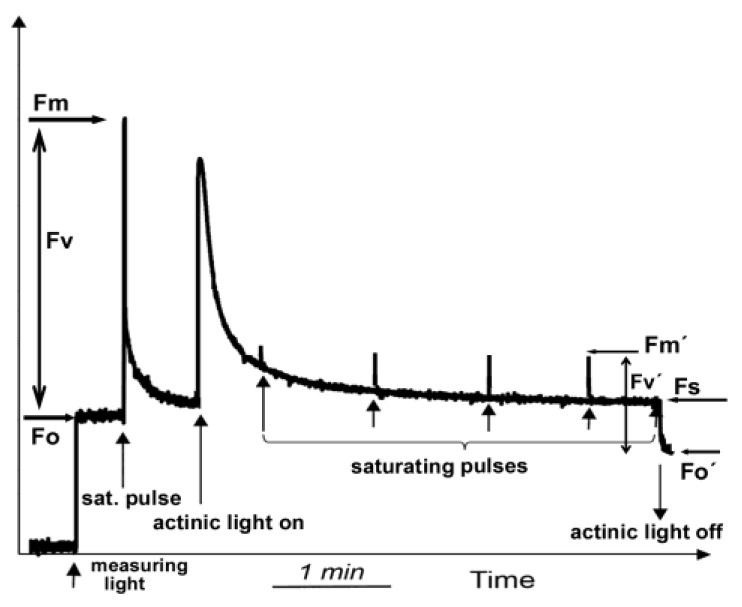
Measuring the basic chlorophyll *a* fluorescence parameters F*o*, F*m*, F*o*′, F*m*′ and F*s* by the pulse amplitude modulation (PAM) method using dark-adapted leaf material. The minimal level of chlorophyll *a* fluorescence (F*o*) in the dark is measured by a low light intensity, called measuring light (ML), that initiates e^−^ transport. A brief saturating pulse of light scores the maximum yield of fluorescence (F*m*) in the dark state. The difference between F*m* and F*o* is the variable fluorescence (F*v*). Under actinic light (AL) illumination, the maximum fluorescence in the light-adapted state (F*m*′) can be estimated with another saturating pulse. The steady-state level of fluorescence (F*s*) in the light is measured immediately before switching off the AL. Immediately after switching off the AL, the minimal level of chlorophyll fluorescence in the light (F*o*′) is measured. The difference between F*m*′ and F*o*′ is the variable fluorescence (F*v*′) in the light (adopted from [32]).

Chlorophyll *a* fluorescence can be detected using various methods, such as the pulse amplitude modulation (PAM) method. Before starting the measurements, the leaf has to be dark-adapted for several minutes, which depends on the light intensity to which the leaf was exposed before measurement and the plant species. The minimal level of chlorophyll *a* fluorescence (F*o*) in the dark (Figure 3) is measured by a low light intensity, called measuring light (ML), while the maximum yield of fluorescence in the dark (F*m*) is evaluated with a saturating light pulse. Under actinic light (AL) illumination, which is the applied light intensity, the maximum fluorescence in the light-adapted state (F*m*′) can be estimated with another saturating pulse (Figure 3). The steady-state level of fluorescence in the light (F*s*) is measured immediately before switching off the chosen AL intensity (Figure 3). After switching off the AL, the minimal level of chlorophyll fluorescence in the light (F*o*′) is measured (Figure 3). The difference between F*m*′ and F*o*′ is the variable fluorescence in the light (F*v*′) (Figure 3). From these measured basic chlorophyll fluorescence parameters, some others can be calculated which are more often used in chlorophyll *a* fluorescence analysis (Table S1 in Supplementary Materials).

Although chlorophyll *a* fluorescence in plants is only 0.6–5%, it offers valuable information about the partitioning of the absorbed light energy at PSII. The absorbed light energy is allocated to PSII photochemistry (Φ*_PSII_*), regulated non-photochemical energy loss in PSII (Φ*_NPQ_*), and non-regulated energy loss in PSII (Φ*_NO_*), that are equal to 1 [18,62]. A pulse-amplitude-modulated (PAM) fluorometer can measure the parameters of the light energy partitioning at PSII and also several other aspects associated with the light reactions of photosynthesis [18].

## 5. Chlorophyll *a* Fluorescence Imaging Analysis

Abiotic or biotic stress conditions may not disturb whole-leaf photosynthesis in a uniform approach [63,64,65,66,67], and thus photosynthetic functioning may be tremendously heterogeneous at the leaf surface [68,69]. Thus, for the evaluation of whole-leaf photosynthetic functioning, standard “point” chlorophyll fluorescence analysis cannot reflect the physiological status of the whole leaf, and the improved method of chlorophyll fluorescence imaging analysis has to be used [33,60,70,71]. Pulse-amplitude-modulated (PAM) fluorescence imaging is based on fluorescence signals from a CCD camera, allowing inspection of spatial heterogeneities in photosynthetic parameters and is considered an evolving tool to evaluate phytotoxic effects under biotic or abiotic stress conditions [33,40,60,72,73]. PAM fluorescence imaging analysis can powerfully assess the fluctuations that appear in the amount of the absorbed light energy and the ways the energy is used [33,60,70]. The absorbed light energy can be either used for photochemistry at PSII (Φ*_PSΙΙ_*), dissipated as heat (Φ*_NPQ_*), or lost by the non-regulated process (Φ*_NO_*) that can lead to ROS generation [33,40,62].

Chlorophyll *a* fluorescence (ChlF) results from the absorbed light energy that is not used for photochemistry or dissipated as heat and it can be interpreted in order to acquire information about the status of the photosynthetic apparatus and especially of PSII [18,56,57,59,60,61]. ChlF parameters can be used to access photosynthetic function and plant tolerance to environmental stresses [31,74,75,76,77,78,79,80,81,82,83,84]. The method is low cost but highly sensitive, as well as rapid and non-destructive [31,59,85,86,87,88,89,90].

## 6. Plant Phenotyping 

Most agricultural land experiences biotic and abiotic stresses that can significantly reduce agricultural yields [91]. Understanding the plant response mechanisms to stress and putting this knowledge into practice are fundamental parts of sustainable agriculture [91]. Numerous imaging techniques have allowed rapid imaging analysis of plant physiological attributes under abiotic and biotic stresses for high-throughput screening [18,92,93,94]. The advent of new imaging techniques has significantly contributed to plant phenotyping, yielding abundant data pertaining to plant physiological status [95]. The selection of appropriate imaging sensors is crucial when designing phenotyping setups, as it depends on the specific research objectives [18,96]. These techniques should enable the early detection of pre-symptomatic changes in plant functional status, long before any visible symptoms appear, facilitating plant tolerance screening [18,92]. By the time visible stress symptoms are detected, the plant may have already experienced significant damage [18,92]. Current imaging techniques allow a non-invasive monitoring of plant physiological status under biotic or abiotic stress [18,60,95,97]. One of the most effective non-invasive imaging techniques for plant health evaluation and stress tolerance monitoring, under abiotic or biotic stress, is chlorophyll *a* fluorescence imaging which assesses spatiotemporal fluctuations in photosynthetic activity across leaves [60,65,92,96,98,99]. Crop sensing by imaging techniques allows the early-stage detection of biotic or abiotic stress to avoid damage and consequently significantly yield losses [3,18,38,58,60,92,100,101,102,103,104,105].

Global climate change is a huge challenge in the agricultural industry in trying to meet the increasing demand for food worldwide [91]. Compared to top yields under perfect situations, the losses associated with abiotic and biotic stress can decrease yields by 65–87% depending on the crop [106]. 

## 7. Chlorophyll Fluorescence Imaging for Abiotic Stress Detection

Different intensities of abiotic stress can trigger diverse plant responses and stimulate distinctive stress defense pathways [38,66,67]. Plant responses to abiotic stress are not linear related to the intensity of the stress [33,38,45,87]. Stress severity can influence plant responses in a hormetic mode [33,38,107], which is also observed in the metabolic responses of plants growing under abiotic stress conditions [108]. Hormesis has been described as the effect of a small-dose or short-duration stressor on an organism that is followed by a destructive effect at a larger dose or longer duration of the same stressor or small-dose- or short-duration-stressor inhibition and larger-dose or longer-duration stimulation [38]. In recent years, chloroplasts have been proposed to be environmental sensors, playing an essential role in plant responses to various abiotic and biotic stresses and participating in plant stress tolerance to environmental changes [109,110,111,112]. Chlorophyll fluorescence imaging analysis is a non-destructive phenotyping method that can predict chloroplast function and responses under optimum [32], or suboptimum [71] growth conditions and estimate photosynthetic tolerance mechanisms to abiotic [75,76,77,113,114,115] or biotic [40,104] stresses. ChlF can detect stresses before visual symptoms develop, which is ideal in screening of genotypes for the early identification of those with high tolerance to abiotic and biotic stress [113].

**Figure 4:**
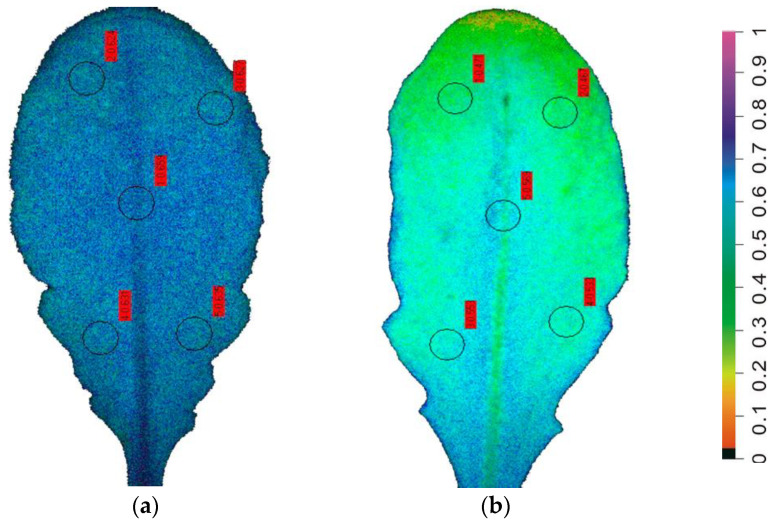
Leaf color-coded pictures of the fraction of open PSII reaction centers (q*p*) from well-watered (control) *Arabidopsis thaliana* plants (**a**), and from *A. thaliana* plants at the onset of water-deficit stress (95–96% of the control plants soil volumetric water content, SWC) (**b**). A color code on the right-side shows q*p* values with a range from 0 to 1 (adopted from [18]).

When the water content of the soil is limited or when transpiration is intensive, plants undergo drought stress [9,18,38]. Chlorophyll *a* fluorescence imaging permits the early-stage detection of drought stress, avoiding damage and consequently significantly yield losses [18,38]. Implementation of chlorophyll *a* fluorescence imaging analysis allowed visualization of leaf spatial photosynthetic heterogeneity at the onset of drought stress (Figure 4), allowing the pre-symptomatic monitoring of early drought stress warning signals in a non-destructive way by providing whole-leaf color pictures of PSII photochemistry [18,38]. The spatial heterogeneity of the fraction of open PSII reaction center (q*p*) images was much higher at the onset of water-deficit stress than in the well-watered (control) *Arabidopsis thaliana* plants (Figure 4).

The demand for increasing crop yield owing to the rising world population and the requests for nutritious food has inevitably led to the uncritical use of chemical fertilizers [116]. The use of ChlF analysis for evaluating physiological disorders triggered by herbicides and pesticides before the appearance of injury symptoms has been proven in many studies [43,117,118,119,120,121,122,123,124]. The principal component analysis of specific ChlF parameters was proposed as a possible species-specific method to sense nutrient deficiencies [125], and for determining the physiological performance of nutritional solutions {magnesium (Mg), phosphorous (P), manganese (Mn), copper (Cu), and iron (Fe)} [126]. Under field conditions, chlorophyll *a* fluorescence analysis provided detection of P deficiency in the critical phase and prevented yield reductions [127]. A correlation was presented between the appearance of the I-step in the OJIP transients, and the phosphorous pool [127]. Diagnosis and remediation of manganese (Mn) deficiency in barley was carried out by utilizing ChlF measurements [128]. The changes that were observed were specific for Mn and did not occur in sulfur- (S), Mg-, Fe- or Cu-deficient plants [128]. ChlF analysis has become a prevalent method to evaluate the impact of various nutrient deficiencies such as nitrogen (N), calcium (Ca), potassium (K), boron (B), S, Mg, P, Mn, and Fe on PSII function [129]. The methods of chlorophyll fluorescence and machine learning were successful in detecting early plant stress that resulted from the combination of nutrient status in natural conditions [130].

The minimum fluorescence (F*o*) that was linked negatively to plant growth was proposed as a valuable indicator to screen rootstocks for root hypoxia stress [131], while F*v*/F*m* proved valuable for estimating the yield performance of wheat under severe drought stress at anthesis [132]. Oláh et al. [72], by assessing several parameters, using chlorophyll *a* fluorescence imaging-based phenotyping, concluded that the light-adapted parameters were more sensitive than the dark-adapted parameters. Yet, they decided that among the dark-adapted parameters, the most-studied parameter, F*v*/F*m*, was shown to be less sensitive and suggested that future studies might consider evaluating F*v*/F*o*, which proved to be more responsive to phytotoxic effects [72]. The parameter F*v*/F*o* has been recommended to be a more suitable parameter than F*v*/F*m* as it has the capability to discriminate between small differences in photosynthetic function [32,133,134,135,136,137]. Furthermore, the parameter F*v*/F*m* has been recently frequently researched in terms of its usefulness [3,68,138,139,140,141,142].

Among abiotic stresses, drought stress was the one most studied by ChlF methods [18,19,68,73,132,138]. Between the ChlF parameters, the redox state of quinone A (Q_A_), that is photochemical quenching (q*p*), was established to be the most suitable indicator, since it was more accurate in evaluating the impact of abiotic stress on plants [18,33,61,143].

**Figure 5:**
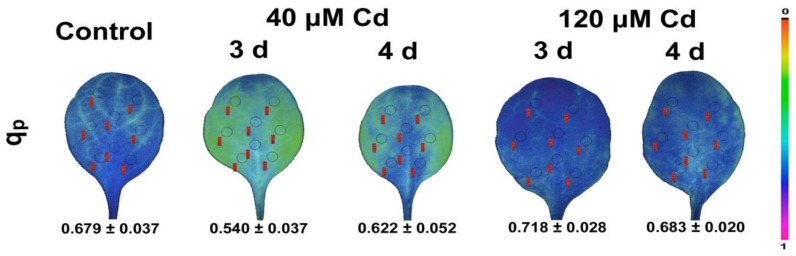
Leaf color-coded pictures of the fraction of open PSII reaction centers (q*p*) from *Noccaea caerulescens* plants grown under 0 (control), 40 or 120 µM Cd^2+^ for 3 and 4 days. A color code on the right-side shows q*p* values with a range from 0.0 to 1.0 (adopted from [69]).

Under elevated CO_2_ experiments, the effective quantum yield of PSII photochemistry (Φ*_PSII_*) was shown by principal component analysis to correlate with water use efficiency, yield, and plant height [144]. Chlorophyll *a* fluorescence imaging analysis revealed the spatial heterogeneity of photosynthetic function under heavy metal stress, e.g., cadmium (Cd) (Figure 5) and zinc (Zn) [87,145]; metal nanoparticles (NPs), e.g., copper (Cu) and Zn [81]; or metal-oxide NPs, e.g., CuO and ZnO [146,147].

**Figure 6:**
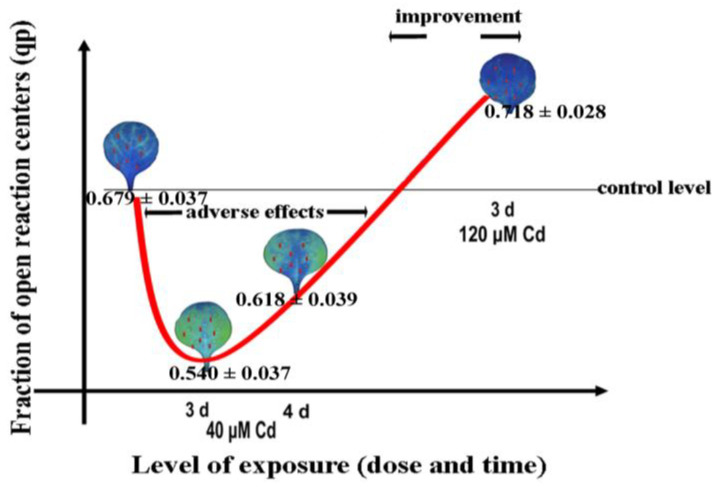
A U-shaped biphasic response curve to Cd exposure of the fraction of open PSII reaction centers (q*p*) in *Noccaea caerulescens*. After 3 days exposure to a 40 µM Cd concentration, a decreased fraction of open PSII reaction centers (q*p*) was observed, while a longer exposure time (4 d) resulted in an increased q*p*, due to the induction of a stress defense response. The same exposure time (3 days) with 120 µM Cd resulted in an increased q*p*. This hormetic response was suggested to be triggered by the increased level of ROS that is considered to be beneficial for triggering defense responses (adopted from [33]).

The hormetic response of the redox state of quinone A (Q_A_), an estimate of the fraction of open PSII reaction centers (q*p*), of *Noccaea caerulescens* plants exposed to cadmium (Cd) stress was induced with 40 µM Cd (Figure 6), due to an increase in ROS generation [33]. A baseline amount of ROS is crucial for sustaining life but under stressful conditions, an elevated level of ROS is regarded as beneficial for initiating defense responses and the acclimation mechanism related to plant stress tolerance [33,34,37,38]. However, an increased level of ROS beyond certain limits can be detrimental to plants [33,34,38,66,148]. 

The non-destructive phenotyping technique of chlorophyll fluorescence imaging was used to study early detection effects of salt stress [149], or nutrient deficiency [150], on photosynthetic traits, as well as examine plant phenotypic trait components associated with the growth and development of different genotypes [151], and also in the selection of biotic- and abiotic-tolerant genotypes for crop improvement [152].

The chlorophyll fluorescence parameters of the effective quantum yield of PSII photochemistry (Φ*_PSII_*), the electron transport rate (ETR), and the redox state of quinone A (Q_A_) or, in other words, the fraction of open reaction centers of PSII (q*p*), were found to be positively correlated with wheat grain yield per plant under water stress [153]. Combining multicolor fluorescence imaging with machine learning was described as a promising phenotyping approach that allows detection of early plant drought stress, offering complete information on the drought stress effects on photosynthesis and secondary metabolism [154]. Stressful environmental conditions not only impact the process of photosynthesis but also exert an influence on the production of secondary metabolites [154].

The spatiotemporal analyses of fluorescence images have given information on the response of plants to environmental stresses [60,63,155,156], and on natural compound-induced phytotoxic stress [157], without causing any damage to plants. Imaging analysis based on color parameters proved to be a consistent prediction method for assessing plant nutrition and crop vegetation status and for agricultural production estimation [158]. Chlorophyll fluorescence analysis is now considered a method of foliar diagnosis for evaluating and monitoring the state of plant nutrition, in order to make improvements in the nutrients provided [158,159].

The chlorophyll *a* fluorescence imaging method was successfully employed for discriminating cold-tolerant and cold-sensitive *Arabidopsis thaliana* accessions [160] as well as for high-throughput sensing of leaf water deficit in six *A. thaliana* accessions [161]. The method of ChlF analysis was successfully applied for studying the impact on the photosynthesis of various abiotic stresses, such as drought, salinity, extreme temperatures, light intensity, nutrient deficiencies, herbicides and heavy metals [145,162]. This method was also used to evaluate different efficient water-saving management methods under different farming modes [163]. Moreover, it is used in agriculture, forestry, ecology, climate change studies, and vegetation research [162]. Recently, maximum quantum yield measurements, accurately depicted changes in grafted plants and served as valuable tools for monitoring graft functionality [164]. 

Chlorophyll *a* fluorescence imaging detection revealed seasonal changes in photosynthetic function and needle photosynthetic heterogeneity [165]. Additionally, F*v*/F*m* images acquired by chlorophyll *a* fluorescence imaging permitted differentiation of two Brassica lines differing in the level of glucosinolates, which are involved in stress responses [166]. ChlF induced by a weak light excitation without dark adaption was used to classify plants as healthy or unhealthy [167].

Screening for the quantum efficiency of PSII in response to increasing temperatures has quantified key aspects of the relationship between PSII efficiency and temperature in grapevines [168], tropical tree species [169], rice [170], and wheat [171]. Such data from ChlF measurements have recently enabled the genetic dissection of photosynthetic heat tolerance in African (*Oryza glaberrima*) and Asian (*Oryza sativa*) rice [172]. Among other plant phenotyping methods, three-dimensional (3D) sensing and hyperspectral imaging were designed for measuring various plant parameters for sensing and quantifying plant traits [173].

## 8. Chlorophyll Fluorescence Imaging for Biotic Stress Detection

Abiotic stresses interact with biotic stresses and this can result in a synergistic action or an antagonistic action. Water-deficit stress, for example, triggers increased plant resistance to herbivores but negatively influences indirect defense and tolerance [174]. However, the interaction between water-deficit stress and herbivory is complex and problematic to simplify [174]. For instance, emerging evidence suggests that plants subjected to drought stress tend to exhibit reduced attractiveness to natural enemies of herbivores, leading to a reduction in the natural enemy community associated with these plants [65,174,175]. Similarly, the plant microbiota that can be beneficial, neutral or detrimental to the plant physiology are affected by abiotic stress, leading to different effects on plant fitness and performance [116].

Promoting sustainable agricultural practices is vital for increased production and providing sustenance to growing populations, with a simultaneous decrease in using chemical fertilizers and pesticides/herbicides [116]. A solution to this is the use of arbuscular mycorrhizal fungi (AMF), or similar beneficial bacteria and/or fungi can that establish mutualistic symbioses with most agricultural crops, and are considered vital tools in the environmentally friendly agriculture [116]. Arbuscular mycorrhizal symbiosis has been shown to increase photosynthetic function and plant growth with the implementation of chlorophyll *a* fluorescence imaging analysis [32].

Global climate change is projected to impact plant–insect interactions and increase crop damage through elevated air temperatures. This effect can be attributed to two primary factors. Firstly, higher temperatures stimulate insect metabolism, leading to intensified crop damage. Secondly, elevated temperatures hinder the plant’s natural cooling mechanism by impeding stomatal opening through herbivore-induced jasmonate signaling. Consequently, leaves experience overheating, reduced photosynthesis and ultimately growth inhibition [104,176].

Plant interactions with pathogens and pests frequently trigger modifications in plant metabolism as part of the plant defense mechanisms to limit nutrient availability to pathogens or as a result of pests manipulating the plant’s metabolism for their own benefit [177]. Interactions of plants with beneficial microbes can also alter the plant response to pathogen infections [116]. The consequences of biotic stresses on leaf plant physiology are usually heterogeneous, both spatially and temporarily, and thus the most appropriate method to evaluate photosynthetic function is the use of chlorophyll fluorescence imaging analysis [64,65,104,177].

Insect herbivory is known to influence photosynthesis negatively [178,179,180] by down-regulating the photosynthesis-related gene expression [181], although reports of a compensatory response of the photosynthetic function in the remaining tissue are not exceptional [104,182,183,184]. Photosynthesis in the undamaged tissue plays a vital role on how the plant will overwhelm herbivory because the energy required for the synthesis of defense response compounds is generated from the light reactions of photosynthesis [185].

The spatiotemporal heterogeneity of PSII photochemistry after short-term biotic stress was quantified by chlorophyll fluorescence imaging analysis that also revealed the existence of a compensatory photosynthetic response mechanism of PSII [104]. Among the ChlF parameters, the redox state of quinone A (Q_A_) revealed the uppermost spatiotemporal heterogeneity, being the most appropriate indicator to evaluate photosynthetic function and also the influence of abiotic and biotic stress on plants [33,104,186]. 

**Figure 7:**
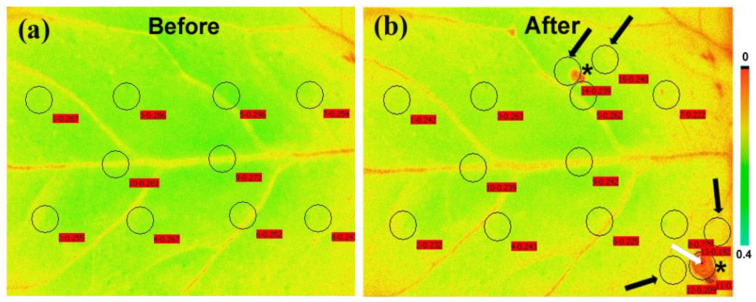
Leaf color-coded pictures in which the color of each pixel represents the level of *Fm* (maximum chlorophyll *a* fluorescence in the dark) in tomato leaflets before (**a**) and after 15 min feeding by *Spodoptera exigua* larvae (**b**). The ten areas of interest (AOIs) before feeding are shown by circles in (**a**), while the same AOIs, together with the two feeding spots (shown by asterisks), and five more AOIs (shown by arrows) were added in (**b**). The white arrow points a feeding spot which covers the whole AOI. Black arrows point out surrounding zones near the existing AOIs. The circles of AOIs are supplemented by red labels with the *Fm* value at their location. The color code on the right-side ranges from pixel values 0 to 0.4 (adopted from [104]).

**Figure 8:**
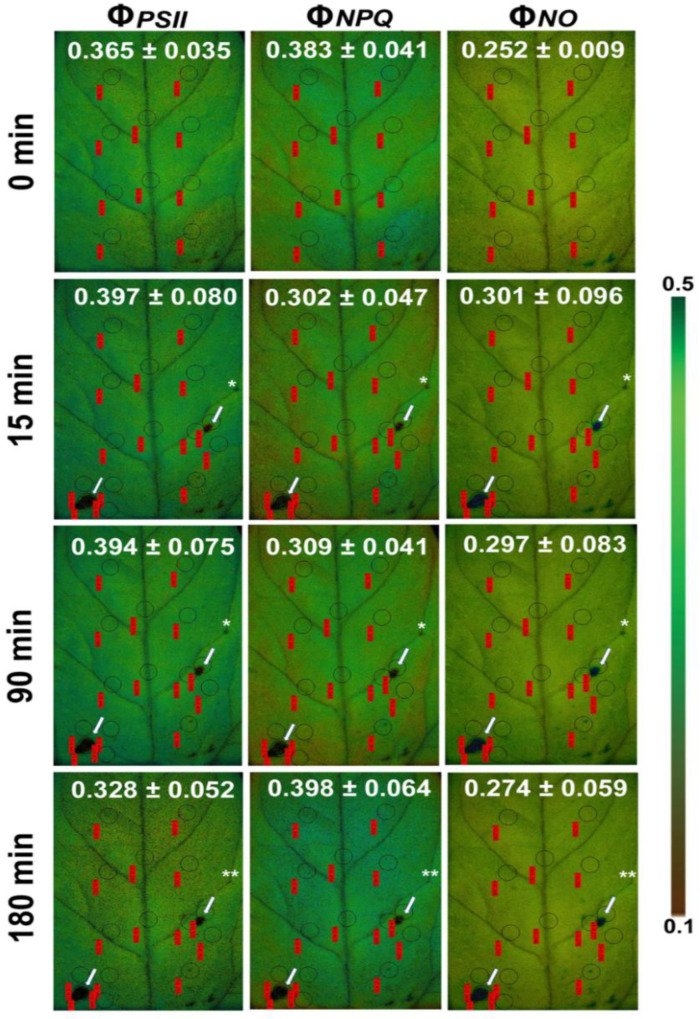
Color-coded pictures of Φ*_PSΙΙ_*, Φ*_NPQ_*, and Φ*_NO_* of a *Solanum lycopersicum* leaflet before (0 min) and after 15, 90, and 180 min feeding by *Spodoptera exigua* larvae. The areas of interest (AOIs) before feeding are shown by circles, while the same AOIs, together with the AOIs of the three feeding spots (shown by asterisk and arrows), and the surrounding to feeding zones AOIs are shown. The circles of AOIs are supplemented by red labels with values of the corresponding parameter. The color code on the right side of the images shows pixel values ranging from 0.1 (dark brown) to 0.5 (dark green) (adopted from [104]).

A decrease in the maximum chlorophyll fluorescence (*Fm*) of tomato leaves, as soon as, 15 min after feeding by the *Spodoptera exigua* larvae was revealed by chlorophyll *a* fluorescence imaging analysis (Figure 7) [104]. A decrease in *Fm* leads to a decline in F*v*/F*m*, which indicates photoinactivation [187]. However, color-coded pictures of the effective quantum yield of PSII photochemistry (Φ*_PSII_*), also obtained by chlorophyll *a* fluorescence imaging, revealed the existence of a compensatory photosynthetic response mechanism that was induced 15 min after *Spodoptera exigua* feeding, at the feeding zone’s surrounding area [104]. Thus, a photosynthetic compensation mechanism was triggered at leaf areas not touched by herbivory as was evident by the increased photochemical quenching (q*p*), and the increased efficiency of PSII reaction centers (F*v*′/F*m*′) [104]. Moreover, the compensatory reaction mechanism at the surrounding area of the feeding zone and at the rest of the leaflet was suggested to be triggered by the singlet oxygen (^1^O_2_) that was formed at the feeding zone [104]. The phenomenon of compensatory photosynthesis in the presence of herbivory can be attributed to an increased requirement for the remaining leaf area to produce larger amounts of carbon. This process necessitates a higher proportion of absorbed light energy for photochemistry [182]. Consequently, plants experiencing higher herbivory levels may develop compensatory mechanisms as an approach to increase fitness under these conditions [182]. The ability of plants to activate this compensatory photosynthetic mechanism depends on the amount of consumed leaf tissue by the herbivore, the timing of herbivory, the feeding style of the herbivore, the environmental conditions, and the plant species [104,182]. The response of tomato PSII photochemistry to *Spodoptera exigua* feeding showed a hormetic response since after 180 min of feeding, Φ*_PSII_*, Φ*_NPQ_*, and Φ*_NO_* returned almost to before feeding levels [104] (Figure 8). The singlet oxygen (^1^O_2_) molecule that was formed at the feeding zone was suggested to act as the signaling molecule that induced the hormetic response [104]. Chloroplasts, which communicate with the nucleus through retrograde signaling, have recently been presented as key regulators of plant responses to biotic and abiotic stress conditions [188]. ^1^O_2_ controls carbon metabolism and a set of nuclear photosynthetic genes, as well as plastid mRNA processing [182,189].

The assessment of disease resistance is thought to be a central aspect of plant phenotyping for an increase in crop yield [190]. By using chlorophyll fluorescence imaging, de Torres Zabala et al. [109] revealed, before bacterial multiplication, the rapid inhibition of photosynthesis in *Arabidopsis thaliana* by *Pseudomonas syringae* and furthermore the mechanism of *P. syringae* action. Measurements of the effective quantum yield of photochemistry (Φ*_PSII_*) at the whole leaf surface, acquired by chlorophyll *a* fluorescence imaging, were used in *Lupinus albus* L. plants to determine the fungal pathogen *Pleiochaeta setosa* 48 h after inoculation [191]. Recently, Suárez et al. [190] also used chlorophyll fluorescence imaging analysis as a tool to identify common bean (*Phaseolus vulgaris* L.) lines that were resistant to pathogens and for the development of a disease susceptibility index. Chlorophyll *a* fluorescence imaging was capable of detecting the increase in the effective quantum yield of photochemistry (Φ*_PSII_*), as soon as 30 min after *Botrytis cinerea* spore suspension application in tomato (*Solanum lycopersicum* L.) leaves [40], and the virulence of 15 isolates of *Botrytis cinerea* on strawberry leaves [192]. The responses of tomato PSII to *B. cinerea* after 30 min [40] and to *Spodoptera exigua* after 15 min feeding [104] indicate a hormetic temporal response in terms of “stress defense response” and “toxicity”, expanding the features of hormesis to biotic factors [104].

## 9. Conclusions

The utilization of existing imaging tools in plant phenotyping holds great potential for accelerating advancements in our understanding of plant functionality. These instruments can establish connections between gene function and environmental responses across multiple pathways, such as metabolic, biochemical, and signaling processes [193]. Among these tools, ChlF analysis stands out as a rapid, non-invasive, cost-effective, and highly sensitive method. This method provides a precise estimate of photosynthetic performance and enables the detection of various stress impacts on plants. The potential of chlorophyll fluorescence imaging as a technique to detect biotic and abiotic stresses before visual symptoms appear has been documented in horticulture and has effectively been applied for diverse purposes, both in preharvest and postharvest situations [13,60,61]. Chlorophyll fluorescence imaging, as a technique, is particularly valuable for examining the heterogeneity of leaf lamina photosynthesis under both biotic and abiotic stress factors, and also for screening of large amounts of samples, providing early stress detection diagnosis. However, further research in this area remains of utmost importance, with the ultimate objective of expediting agriculture production. The method of chlorophyll fluorescence analysis can be used to construct comprehensive stress tolerance databases for various crop varieties in order to optimize photosynthetic function for climate change in terms of environmental conditions that will enable increased crop productivity.

Among the diverse ChlF parameters, the fraction of open PSII reaction centers (q*p*), has been proposed as the most suitable indicator for early stress detection. Recent studies have consistently found q*p* to be highly sensitive and well suited for probing photosynthetic function, enabling the early-stage assessment of the impact of abiotic and biotic stresses on plants [18,39,63,143,194,195]. We recommend that in further research, scientists consider including in their work the chlorophyll fluorescence parameter of the redox state of Q_A_.

## Data Availability

The data presented in this study are available in this article.

## Supplementary Materials

The following supporting information can be downloaded at: https://www.mdpi.com/article/10.3390/bios13080796/s1, Table S1: Definitions of the most common used chlorophyl fluorescence parameters.
