# Supplementary material for: Early-Stage Detection of Biotic and Abiotic Stress on Plants by Chlorophyll Fluorescence Imaging Analysis
Source: Biosensors (Basel). 2023 Aug 8;13(8):796. doi: 10.3390/bios13080796 (PMC10452221; doi:10.3390/bios13080796)
Supplement: Supplementary file 1 [file biosensors-13-00796-s001.zip › biosensors-2496945-supplementary.pdf]

biosensors

MDPI

Early-Stage Detection of Biotic and Abiotic Stress on Plants by Chlorophyll Fluorescence Imaging Analysis

Julietta Moustaka and Michael Moustakas

Table S1. Definitions of the most common used chlorophyl fluorescence parameters

Parameter	Definition	Calculation
Fv/Fm	Maximum efficiency of PSII photochemistry	$(Fm - Fo)/Fm$
Φ_{PSII}	Effective quantum yield of PSII photochemistry	$(Fm' - Fs)/Fm'$
Φ_{NPQ}	Quantum yield of regulated non-photochemical energy loss in PSII	$Fs/Fm' - Fs/Fm$
Φ_{NO}	Quantum yield of nonregulated energy loss in PSII	Fs/Fm
Fv'/Fm'	Efficiency of PSII reaction centers	$(Fm' - Fo')/Fm'$
Fv/Fo	Efficiency of the oxygen evolving complex (OEC) on the donor side of PSII	$(Fm - Fo)/Fo$
ETR	Electron transport rate	Φ_{PSII} × PAR × c × abs, where PAR is the photosynthetically active radiation, c is 0.5, and abs is the total light absorption of the leaf taken as 0.84
qp	Photochemical quenching, representing the redox state of quinone A (Q_A), or in other words the fraction of PSII reaction centers in open state	$(Fm' - Fs)/(Fm' - Fo')$
NPQ	Non-photochemical quenching reflecting the dissipation of excitation energy as heat	$(Fm - Fm')/Fm'$
EXC	Excess excitation energy	$(Fv/Fm - \Phi_{PSII})/Fv/Fm$
1-qp	Excitation pressure	$1 - [(Fm' - Fs)/(Fm' - Fo')]$
